# Disruption of the VAPB-PTPIP51 ER-mitochondria tethering proteins in post-mortem human amyotrophic lateral sclerosis

**DOI:** 10.3389/fcell.2022.950767

**Published:** 2022-08-16

**Authors:** Naomi Hartopp, Dawn H W. Lau, Sandra M. Martin-Guerrero, Andrea Markovinovic, Gábor M. Mórotz, Jenny Greig, Elizabeth B. Glennon, Claire Troakes, Patricia Gomez-Suaga, Wendy Noble, Christopher C.J. Miller

**Affiliations:** Department of Basic and Clinical Neuroscience. Institute of Psychiatry, Psychology and Neuroscience, King’s College London, London, United Kingdom

**Keywords:** VAPB, PTPIP51, endoplasmic reticulum, mitochondria, amyotrophic lateral sclerosis

## Abstract

Signaling between the endoplasmic reticulum (ER) and mitochondria regulates many neuronal functions that are perturbed in amyotrophic lateral sclerosis (ALS) and perturbation to ER-mitochondria signaling is seen in cell and transgenic models of ALS. However, there is currently little evidence that ER-mitochondria signaling is altered in human ALS. ER-mitochondria signaling is mediated by interactions between the integral ER protein VAPB and the outer mitochondrial membrane protein PTPIP51 which act to recruit and “tether” regions of ER to the mitochondrial surface. The VAPB-PTPI51 tethers are now known to regulate a number of ER-mitochondria signaling functions. These include delivery of Ca^2+^ from ER stores to mitochondria, mitochondrial ATP production, autophagy and synaptic activity. Here we investigate the VAPB-PTPIP51 tethers in post-mortem control and ALS spinal cords. We show that VAPB protein levels are reduced in ALS. Proximity ligation assays were then used to quantify the VAPB-PTPIP51 interaction in spinal cord motor neurons in control and ALS cases. These studies revealed that the VAPB-PTPIP51 tethers are disrupted in ALS. Thus, we identify a new pathogenic event in post-mortem ALS.

## Introduction

Amyotrophic lateral sclerosis (ALS) is the most common form of motor neuron disease and involves progressive loss of motor neurons resulting in muscle wasting and ultimately paralysis. ALS is now known to be clinically, pathologically and genetically linked to frontotemporal dementia (FTD). FTD is the second most common cause of presenile dementia after Alzheimer’s disease (Ling et al., 2013; Robberecht and Philips, 2013). Thus, many FTD patients display clinical ALS features and likewise many ALS patients develop clinical symptoms of FTD (Ringholz et al., 2005; Wheaton et al., 2007). Both diseases can display similar pathological phenotypes and notably, the accumulation of abnormal aggregates of TAR DNA-binding protein 43 (TDP43) in affected neurons (Arai et al., 2006; Neumann et al., 2006). Finally, both diseases have strong genetic components and mutations in the same genes can cause dominant familial inherited forms of ALS and FTD. Mutant genes causing both ALS and FTD include *TARDBP* encoding TDP43, *FUS* encoding fused in sarcoma and *C9orf72*; the disease causing mutations in *C9orf72* involve expansion of an intronic hexanucleotide repeat which is translated into neurotoxic dipeptide repeat proteins (DPRs) (Ling et al., 2013; Robberecht and Philips, 2013; Abramzon et al., 2020).

There are no cures or effective disease modifying treatments for ALS. Developing new therapies can include correcting damaged molecular, cellular and physiological processes but this is complicated as a large number of changes are seen in ALS. Thus, damage to mitochondria, the endoplasmic reticulum (ER), Ca^2+^ signaling, lipid metabolism, axonal transport, autophagy and inflammatory responses are all features of ALS (Paillusson et al., 2016; Lau et al., 2018; Dafinca et al., 2021; Markovinovic et al., 2022). The biological conundrum is how so many apparently disparate physiological processes are perturbed collectively. The therapeutic challenge is selecting which of these different perturbed processes to prioritize for drug discovery.

Recently, attention has focussed on signaling between the ER and mitochondria and this is because ER-mitochondria signaling regulates many of the functions that are damaged in ALS (Paillusson et al., 2016; Csordas et al., 2018; Lau et al., 2018; Dafinca et al., 2021; Markovinovic et al., 2022). ER-mitochondria signaling involves close contacts between the two organelles (up to approximately 30 nm distances) and the regions of ER in contact with mitochondria are termed mitochondria associated ER membranes (MAM) (Paillusson et al., 2016; Csordas et al., 2018; Lau et al., 2018; Dafinca et al., 2021; Markovinovic et al., 2022). The mechanisms by which ER membranes are recruited to the mitochondrial surface are not fully understood but it is widely accepted that the process involves “tethering proteins” which act to scaffold the two organelles in close proximity. One well characterised tether involves an interaction between the integral ER protein, vesicle-associated membrane protein-associated protein B (VAPB) and the outer mitochondrial membrane protein, protein tyrosine phosphatase interacting protein-51 (PTPIP51) (also known as regulator of microtubule dynamics-3 and family with sequence similarity 82 member A2) (De Vos et al., 2012; Stoica et al., 2014). The VAPB-PTPIP51 tethers are known to control a number of ER-mitochondria regulated functions including inositol 1,4,5-trisphosphate (IP3) receptor delivery of Ca^2+^ from ER stores to mitochondria, mitochondrial ATP production, autophagy, phospholipid synthesis and synaptic activity (De Vos et al., 2012; Stoica et al., 2014; Galmes et al., 2016; Stoica et al., 2016; Gomez-Suaga et al., 2017; Paillusson et al., 2017; Gomez-Suaga et al., 2019; Puri et al., 2019; Yeo et al., 2021; Gomez-Suaga et al., 2022). Loss of synaptic activity is a key feature of ALS and other neurodegenerative diseases (Herms and Dorostkar, 2016; Spires-Jones et al., 2017).

Such findings have prompted investigations into the VAPB-PTPIP51 tethers in ALS and this has revealed them to be disrupted in cell and transgenic mouse models involving mutant TDP43, FUS, and *C9orf72* (Stoica et al., 2014; Stoica et al., 2016; Gomez-Suaga et al., 2022). However, as yet there is no evidence that the VAPB-PTPIP51 interaction is altered in human ALS patients. This is an important omission. Firstly, because transgenic mouse and cell models of ALS do not always fully recapitulate human disease; for example some *C9orf72* transgenic mouse models display hippocampal rather than motor neuron loss (Jiang et al., 2016). More importantly, if correcting disrupted ER-mitochondria signaling and VAPB-PTPIP51 tethering is to be a valid drug target for ALS, it is essential we know whether these features are actually damaged in human disease. Here we address this issue by examining the ER-mitochondria tethering proteins VAPB and PTPIP51 in post-mortem ALS and control tissues.

## Materials and methods

### Antibodies

The following primary antibodies were used in this study: Rabbit and rat antibodies to VAPB and PTPIP51 were as described (De Vos et al., 2012). Rabbit anti-PTPIP51 antibody (Anti-RMDN3, HPA009975) and rabbit anti-IP3 receptor type-3 (HPA003915) were from Atlas Antibodies. Rabbit anti-voltage-dependent anion channel-1 (VDAC1) (ab14734) was from Abcam. Mouse anti-neuron specific enolase (NSE) (BBS/NC/VI-H14 -M0873) was from Dako.

### Human tissues

Post-mortem human spinal cord samples from control and clinically and pathologically confirmed cases of ALS were obtained from the London Neurodegenerative Diseases Brain Bank, King’s College London. All tissue collection and processing were carried out under the regulations and licensing of the Human Tissue Authority, and in accordance with the Human Tissue Act, 2004. The ALS cases analysed all contained TDP43 positive inclusions.

### SDS-PAGE and immunoblotting

Frozen human lumbar spinal cord tissues were prepared for SDS-PAGE as described previously (Lau et al., 2020). Protein concentrations were determined using a bicinchoninic acid protein concentration assay kit (Pierce) according to the manufacturer’s instructions and samples stored at −80°C until required. Samples were separated by SDS-PAGE using Novex 4–12% Tris-glycine gels (Invitrogen) and transferred to Protran nitrocellulose membranes (0.45 μm pore; G.E. Healthcare) using an Invitrogen X-Cell blot II transfer system. After transfer, membranes were blocked in Tris–HCl-buffered saline (TBS, pH 7.3), 0.1% (v/v) Tween-20 containing either 5% (w/v) BSA (for probing for and IP3 receptor type-3) or 5% (w/v) non-fat dried milk powder (for probing all other proteins). Membranes were incubated with primary antibodies in blocking buffer overnight at 4°C, washed and incubated with secondary antibodies and then processed for chemiluminescent detection using a 1:1 dilution of ECL blotting reagents 1 and 2 (GE Healthcare). Chemiluminescent signals were detected with a Bio-Rad ChemiDoc imaging system and analysed using ImageJ; protein signals were normalised to NSE signals from the same sample. Atlas PTPIP51 antibody was used for immunoblots. All primary antibodies were used at 1:2000 apart from anti-NSE which was used at 1:10000 concentration.

### Proximity ligations assays (PLAs) and microscopy

VAPB-PTPIP51 PLAs were performed on 7 μm sections of paraffin wax embedded formalin fixed post-mortem human lumbar spinal cord tissues using rabbit VAPB and rat PTPIP51 antibodies essentially as described previously for studies of post-mortem Alzheimer’s disease tissues, and using Duolink *In Situ* Detection Brightfield kits (Sigma) (Lau et al., 2020). Donkey anti-rabbit *in situ* PLA probes were purchased directly; donkey anti-rat PLA probes were prepared using Duolink *In Situ* Probemaker kits (Sigma). Primary antibodies were used at 1:200 concentration. Following PLAs, sections were counterstained with haematoxylin, dehydrated in graded alcohols and xylene, and mounted using DPX mounting reagent. Experimental controls to demonstrate specificity of the PLAs involved omission of VAPB, PTPIP51 or both VAPB and PTPIP51 antibodies.

Sections were imaged using an Olympus VS.120 slide scanner using an Olympus 40x UPlanSApo NA 0.95 lens and driven by Olympus L100 VS-ASW software. Motor neurons were identified by morphology. They are located in the anterior horn of the lumbar spinal cord and are the largest cells in the spinal cord so are easily identified. Images were analysed as previously described using Visiopharm 2018.4 Image Analyses software with an analyse package protocol created with Author Module (Lau et al., 2020). Briefly, the perimeter of each motor neuron was marked which enabled the relative area and the number of PLA dots within each cell to be calculated by the software.

### Statistical analyses

Statistical analysis was performed using Excel (Microsoft Corporation) and Prism software (version 9; GraphPad Software Inc.). Statistical significance was determined as described in the figure legends. Correlation analyses were performed as previously described (Lau et al., 2020). Briefly, VAPB-PTPIP51 PLA dot numbers per case were correlated with age and post-mortem delay by generating correlation coefficients and significance was established using parametric, two-tailed Pearson tests.

## Results

### VAPB levels are reduced in post-mortem ALS spinal cord

Firstly, we investigated the levels of key ER-mitochondria signaling proteins in post-mortem control and ALS spinal cord tissues. We studied VAPB and PTPIP51 since they function to tether ER domains with mitochondria so as to permit signaling, and IP3 receptor and VDAC1 since they represent the major channel for delivery of Ca^2+^ from ER stores to mitochondria; ER-mitochondria Ca^2+^ exchange controls several functions perturbed in ALS such as mitochondrial ATP production, autophagy and synaptic activity (De Vos et al., 2012; Stoica et al., 2014; Stoica et al., 2016; Gomez-Suaga et al., 2017; Paillusson et al., 2017; Csordas et al., 2018; Gomez-Suaga et al., 2019; Puri et al., 2019; Gomez-Suaga et al., 2022; Markovinovic et al., 2022). There are 3 isoforms of IP3 receptor (type−1, −2 and −3) which all function to transport Ca^2+^ to mitochondria (Bartok et al., 2019). These isoforms show different expression patterns in the nervous system. IP3 receptor type-1 is highly expressed in neurons in the cortex, hippocampus and cerebellum, IP3 receptor type-2 is primarily expressed in glia, and IP3 receptor type-3 is the major isoform in brain stem and spinal cord, including motor neurons, but is largely absent in cortex and hippocampus (De Smedt et al., 1994; Sharp et al., 1999; Watanabe et al., 2016). We therefore studied IP3 receptor type-3 levels.

**FIGURE 1:**
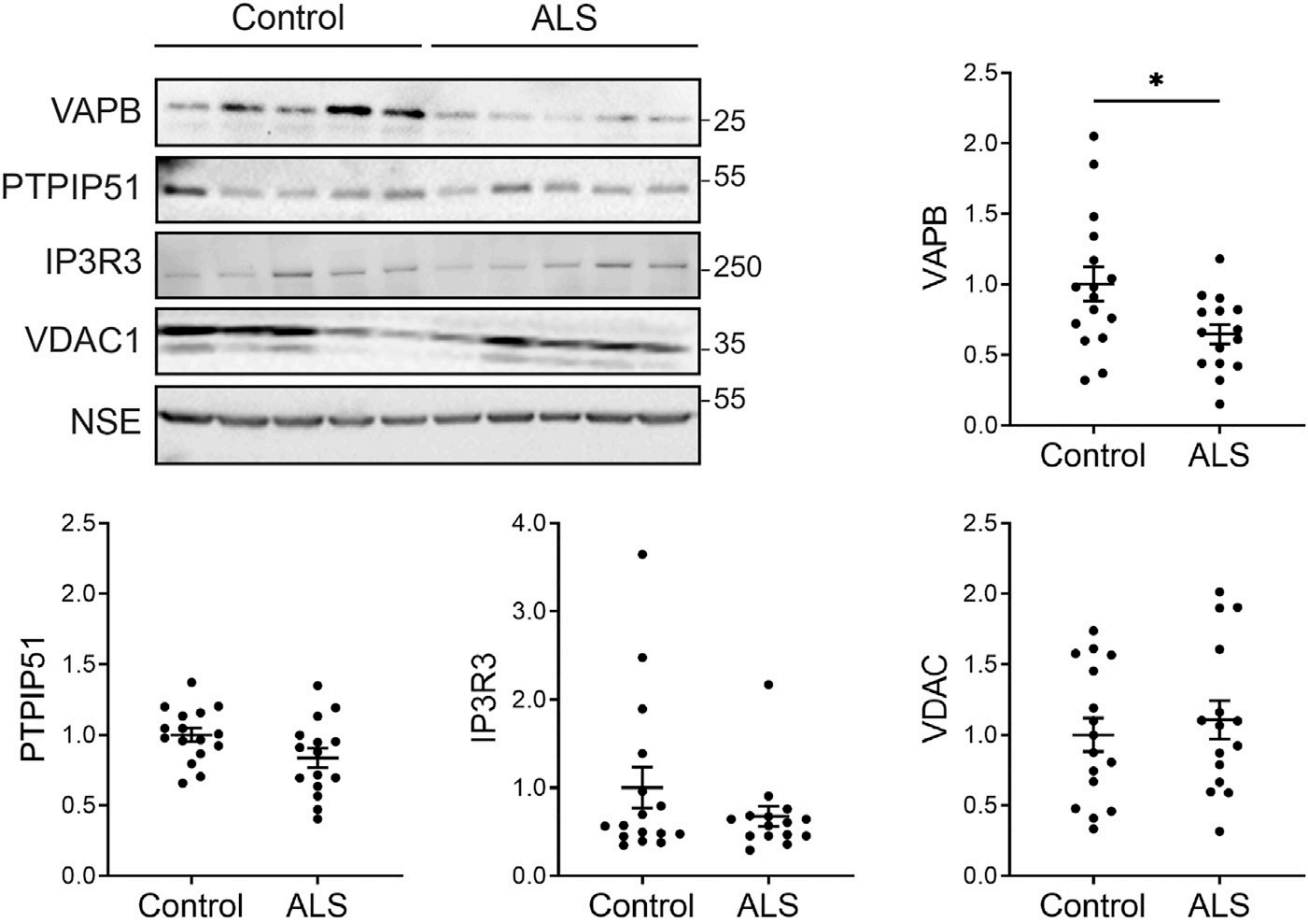
Expression of VAPB, PTPIP51, IP3 receptor type-3 and VDAC1 proteins in post-mortem control and ALS spinal cords. Representative immunoblots are shown. Graphs show quantification of protein levels in the different samples following normalisation to NSE levels in the same sample. N = 16 control and 15 ALS cases. Data were analysed by unpaired *t*-test. Error bars are standard error of means (s.e.m.); *p < 0.05.

To quantify the levels of these proteins, we performed immunoblots of post-mortem control and ALS spinal cord tissues. Details of these human cases are shown in Table 1 and involve tissues from 16 control and 15 ALS patients. The levels of each protein were normalised to the levels of NSE as described by others in similar studies of human post-mortem neurodegenerative disease tissues (Tiwari et al., 2015; Kurbatskaya et al., 2016; Lau et al., 2016; Tiwari et al., 2016; Morotz et al., 2019a; Morotz et al., 2019b; Lau et al., 2020). Compared to controls, VAPB levels were significantly reduced in the ALS tissues but there were no changes in the levels of PTPIP51, IP3 receptor type-3 or VDAC1 (Figure 1).

**TABLE 1:** Data for human post-mortem samples.

Group	Sex	Age	Post-mortem delay (hrs)
Control	M	105	25
Control	F	73	27
Control	F	77	21
Control	M	84	53
Control	F	92	22.5
Control	M	85	55
Control	M	63	23
Control	F	99	32
Control	M	78	24
Control	M	82	24
Control	F	92	9.0
Control	M	97	44
Control	F	84	34
Control	F	89	41
Control	M	81	18
Control	M	79	47
ALS	M	68	78
ALS	M	57	94
ALS	M	69	52.5
ALS	M	68	73
ALS	F	73	70
ALS	F	90	34
ALS	F	59	74
ALS	F	72	53
ALS	M	54	69
ALS	M	77	66
ALS	M	76	51
ALS	F	80	36.5
ALS	F	69	64
ALS	M	73	41.5
ALS	M	71	58

### The VAPB-PTPIP51 interaction is disrupted in spinal cord motor neurons in post-mortem ALS

To determine whether the VAPB-PTPIP51 interaction is disrupted in human ALS, we used *in situ* PLAs to quantify their binding in spinal cord motor neurons in the control and ALS tissues. The distances detected by PLAs are up to about 30 nm which makes these assays suitable for quantifying ER-mitochondria contacts (Soderberg et al., 2006; Paillusson et al., 2016). PLAs including ones for VAPB and PTPIP51 have already been used to quantify ER-mitochondria contacts and signaling in models of ALS, Parkinson’s disease and Alzheimer’s disease (De Vos et al., 2012; Hedskog et al., 2013; Bernard-Marissal et al., 2015; Stoica et al., 2016; Paillusson et al., 2017; Gomez-Suaga et al., 2019; Gomez-Suaga et al., 2022). Most recently, such studies have been extended to analyses of the VAPB-PTPIP51 interaction in human post-mortem Alzheimer’s disease brains (Lau et al., 2020).

**FIGURE 2:**
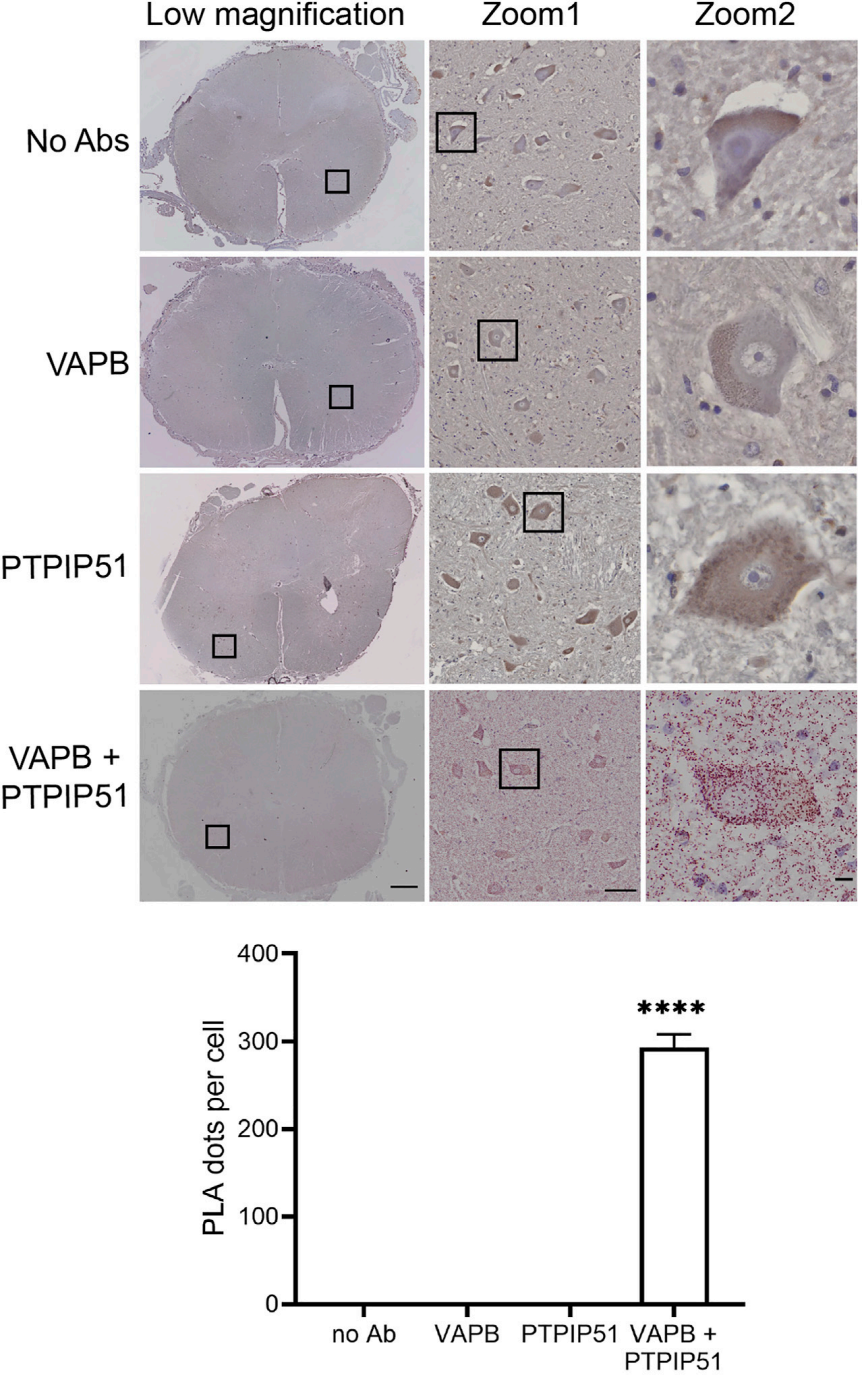
Control experiments demonstrating the specificity of VAPB-PTPIP51 PLAs on human post-mortem spinal cord tissues. Controls involved omission of VAPB, PTPIP51, or both VAPB and PTPIP51 primary antibodies (no primary Ab). The graph shows the number of PLA dots per spinal cord motor neuron in the different experiments. Data were analysed by ANOVA and Tukey post hoc test. N = 20–106 per condition, error bars are s. e.m.; ****p < 0.0001. Scale bars: 1,000 μm (Low magnification), 100 μm (Zoom 1) and 10 μm (Zoom 2).

Firstly, we demonstrated the specificity of the PLAs in control experiments where primary VAPB and/or PTPIP51 antibodies were omitted. Such omission produced none or only very few signals whereas inclusion of both antibodies generated significant positivity (Figure 2). These findings are in agreement with several previous studies including studies of human post-mortem Alzheimer’s disease brains (De Vos et al., 2012; Stoica et al., 2016; Lau et al., 2020).

**FIGURE 3:**
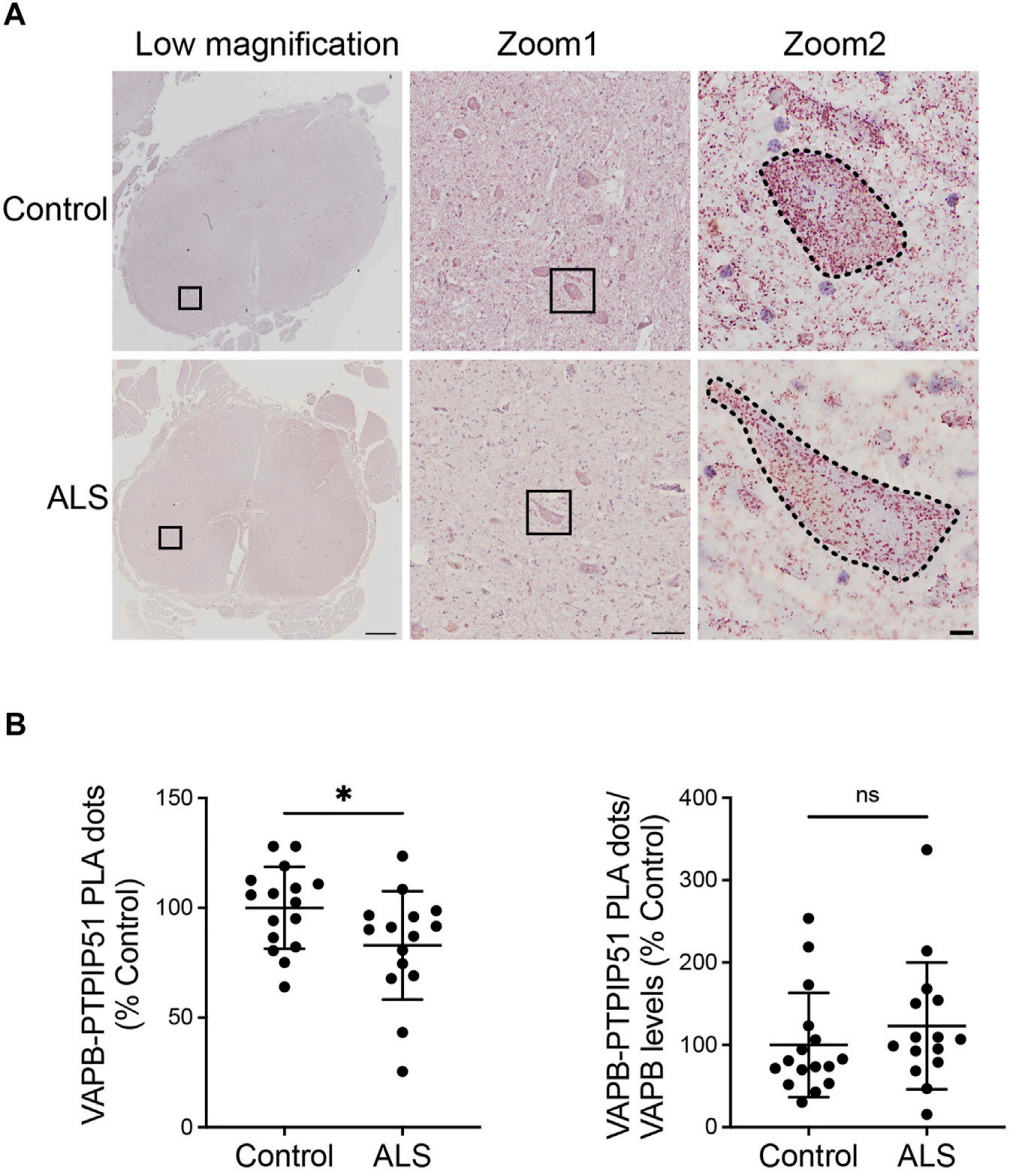
The VAPB-PTPIP51 interaction is reduced in ALS spinal cord motor neurons. **(A)** Representative images of VAPB-PTPIP51 PLAs in control and ALS tissues. Low magnification and two zoom images are shown for each sample; motor neurons are outlined in the highest zoom images. The graph shows the mean number of VAPB-PTPIP51 PLA dots per motor neuron for each case. VAPB-PTPIP51 PLA numbers were normalised to the area of each cell so as to correct for any changes in neuron size in the ALS cases as described in Materials and Methods. **(B)** Graph showing mean numbers of VAPB-PTPIP51 PLA dots following normalisation to VAPB protein levels. N = 16 control and 15 ALS cases. Data were analysed by unpaired *t*-test; Error bars are s. e.m., *p < 0.05, ns not significant. Scale bars; 1,000 μm (Low magnification), 100 μm (Zoom 1) and 10 μm (Zoom 2).

We then quantified the VAPB-PTPIP51 PLA dots in the motor neurons of the 16 control and 15 ALS spinal cords. The number of PLA positive dots per cell were normalised to the area of each cell so as to correct for any changes in neuron size in the ALS cases. Thus, any difference in PLA signal detected in the ALS motor neurons cannot be the consequence of changes in cell size. These studies revealed that compared to controls, VAPB-PTPIP51 PLA signal numbers/cell were significantly reduced in ALS motor neurons (Figure 3A). We also performed correlation analyses to determine whether the number of PLA dots correlated with post-mortem delay or age in the cases studied. These analyses revealed that there were no significant correlations between the number of PLA dots and either of these parameters (post-mortem delay, *r* = −0.102, *p* = 0.58; age *r* = −0.112, *p* = 0.55).

Finally, since we detected a significant reduction in VAPB protein levels in the ALS cases, we analysed the impact of this reduction on the VAPB-PTPIP51 interaction by normalising the VAPB-PTPIP51 PLA dot numbers to VAPB protein levels. The significant reduction in VAPB-PTPIP51 PLA dots in the ALS cases was lost following this normalisation (Figure 3B). This suggest that loss of VAPB may contribute to the reduced VAPB-PTPIP51 interaction in ALS.

## Discussion

A number of studies have now shown that ER-mitochondria signaling is perturbed in ALS (Lautenschlager et al., 2013; Stoica et al., 2014; Bernard-Marissal et al., 2015; Dafinca et al., 2016; Gregianin et al., 2016; Stoica et al., 2016; Watanabe et al., 2016; Tadic et al., 2017; Dafinca et al., 2020; Dafinca et al., 2021; Gomez-Suaga et al., 2022). However, these studies largely focused on experimental models and to date, there is little evidence that ER-mitochondria contacts and signaling are altered in human ALS patient tissues. Here, we utilised PLA technology to study the VAPB-PTPIP51 interaction in post-mortem ALS spinal cord motor neurons.

Our analysis involved 16 control and 15 ALS cases and so represents a highly powered study. Compared to controls, we detected a significant decrease in VAPB-PTPIP51 PLA signals in the ALS motor neurons. Studies of induced pluripotent stem cell neurons derived from patients carrying pathogenic TDP43 and C9orf72 mutations also support a role for perturbation to ER-mitochondria signaling in ALS and this includes disruption to the VAPB-PTPIP51 interaction (Dafinca et al., 2016; Dafinca et al., 2020; Dafinca et al., 2021; Gomez-Suaga et al., 2022). Our findings thus complement and extend these prior studies.

The mechanisms that underlie the disruption to the VAPB-PTPIP51 tethers in ALS are not clear. Clearly the expression levels of VAPB and PTPIP51 affect their interaction and this in turn has been shown to influence ER-mitochondria contacts and linked functions. Thus, siRNA loss of VAPB and/or PTPIP51 reduce ER-mitochondria contacts, IP3 receptor mediated delivery of Ca^2+^ to mitochondria, and downstream functions of this Ca^2+^ delivery (De Vos et al., 2012; Stoica et al., 2014; Gomez-Suaga et al., 2017; Gomez-Suaga et al., 2019). Our finding that VAPB levels are reduced in ALS spinal cord suggests that this loss may contribute to the decrease in the VAPB-PTPIP51 interaction we detect in ALS. Interestingly, others have also reported decreased levels of VAPB in ALS post-mortem tissues (Anagnostou et al., 2010). Indeed, we found that following normalisation of VAPB-PTPIP51 PLA dot numbers to VAPB protein levels, the reduction in the VAPB-PTPIP51 interaction we detected in the ALS cases was lost. This supports the notion that loss of VAPB contributes to the reduced VAPB-PTPIP51 interaction in ALS. Interestingly, there is evidence that VAPB may have other functions aside from ER-mitochondria tethering and that it may act to tether regions of ER with other organelles (Kors et al., 2022). Loss of VAPB may contribute to ALS via mechanisms other than ER-mitochondria tethering.

An alternative possibility is that the ALS linked perturbation of the VAPB-PTPIP51 tethers is linked to activation of glycogen synthase kinase-3β (GSK3β). GSK3β is a negative regulator of the VAPB-PTPIP51 interaction and its activation has been linked to disruption of ER-mitochondria tethering and signaling by ALS mutant TDP43, FUS, and C9orf72 in experimental models (Stoica et al., 2014; Stoica et al., 2016; Gomez-Suaga et al., 2022). However, studying GSK3β activity in post-mortem human tissues is difficult and similar analyses of GSK3β in post-mortem Alzheimer’s disease brains have generated highly conflicting data with some reporting increased and some decreased activity in Alzheimer’s disease (Ferrer et al., 2002; Swatton et al., 2004; Leroy et al., 2007). Such studies have led to the conclusion that it is technically difficult, if not impossible, to measure GSK3β enzymatic activity in post-mortem neurodegenerative disease tissues (Hooper et al., 2008). Analyses of ALS tissues with very short post-mortem times may assist such analyses in future studies.

Whatever the precise mechanisms, our findings demonstrate, for the first time, that the VAPB-PTPIP51 ER-mitochondria tethers are perturbed in human ALS post-mortem motor neurons. As such, they provide clinical support for prior experimental studies which highlighted the role of damaged ER-mitochondria signaling in ALS.

## Data Availability

The raw data supporting the conclusions of this article will be made available by the authors, without undue reservation.
